# 

**Published:** 1992-03

**Authors:** 


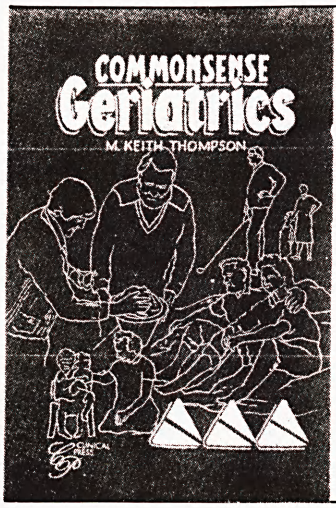

### Commonsense GERIATRICS

**Keith Thompson, General Practitioner, London**
**Examiner for the Diploma in Geriatric Medicine**

150 pages. Papercover. Fully illustrated. ISBN 1-85457-007-2. £12.50

*Clinical Press*

### REGAINING BLADDER CONTROL

*Second edition*

*Clinical Press*

Eileen Montgomery, MCSP.,

For those suffering from incontinence on exertion or following pelvic surgery.

This important patient handbook contains a planned series of exercises aimed at restoring control of the bladder. The anatomical causes of incontinence are clearly explained and illustrated. The book also deals with correct breathing and a balanced diet which will not lead to constipation or to being overweight.

Surgeons, doctors and physiotherapists will be able to recommend this booklet to their patients.

28 pages. Soft cover. 11 line drawings. ISBN: 1 85457 003 X

Price inclusive postage and packing £2.50 per copy or £20.00 per pack of 10 copies.

Please order from your usual bookseller or direct from Clinical Press Ltd., c/o Gazelle Book Services, Falcon House, Queen Square, Lancaster, LA1 1RN, UK.

### THE CHESTERFIELD NUFFIELD HOSPITAL

### Your own private hospital

*Benefit from the experience...!*

For further details contact The Chesterfield Nuffield Hospital

3 Clifton Hill, Clifton, Bristol BS8 1BP. Telephone (0272) 730391

Nuffield Hospitals

**Editorial Committee**
Chairman of Editorial Board, Professor John Farndon
Editor—Dr Paul Goddard
Assistant Editors—Mr M. G. Wilson, Dr Alison Round
Dr Julian Kabala, Dr James Catterall
Secretary—Mr Clive Ward
Member—Dr Kenneth Southgate

**Editorial Office**—*Redland Green Farm, Redland, Bristol BS6 7HF*

**Correspondents**
Dr Andrew Adam (Taunton)
Dr David Levine (Truro)
Dr Jack Davies (Bristol)
Professor Denis Pereira Gray (Exeter)
Dr Jose Jancar (Bristol)
Dr Michael Oliver (Barnstaple)
Mr Michael Reed (Gloucester)
Dr Michael Whitfield (Bristol)

**Associated Societies**
Bristol Medico-Chirurgical Society
Clinical Society of Bath
Severn Faculty of the Royal College of General Practitioners
South Wales, South West and Wessex Rheumatology Club
South West of England and Wales Society of Dermatology
South West British Geriatric Society
South West Obstetricians' and Gynaecologists' Club
South West Orthopaedic Club
South West Paediatric Club
South West Radiologists' Association
South West Urologists' Club
South West Urological Oncology Group
South West Vascular Surgeons
Surgical Club of South West England
Tamar Faculty of the Royal College of General Practitioners
Wessex Association of Clinical Pathologists
West Country Chest Physicians' Club
West Country Physicians' Club

Members of the above organisations receive the journal as part of their membership subscription.
Annual Subscription Rate £25.00 including surface postage.

**Published by**
Clinical Press,
*Redland Green Farm,*
*Redland Green, Redland, Bristol BS6 7HF.*
*Tel 0272 237709/420256*

**Typesetting and Printing by**
*H. E. ILES (Central Press) LTD.,*
*49/51 Downend Road,*
*Kingswood, Bristol BS15 1SF.*
*Tel 0272 614161 (4 lines)*

**THE WEST OF ENGLAND MEDICAL JOURNAL acknowledges with gratitude the help it receives from NUFFIELD HOSPITALS**

While the advice and information in this journal is believed to be true and accurate at the time of its going to press, neither the authors, the editors, nor the publisher can accept any legal responsibility for any errors or omissions that may be made. The publisher makes no warranty, express or implied, with respect to the material contained herein.

### WEST OF ENGLAND MEDICAL JOURNAL

1883—1991 Vols 1—104

### GPs! IF YOU VALUE YOUR TIME YOU WILL VALUE THE. . . .

### 1992 MEDICAL ANNUAL

### 108th issue

### The Yearbook of General Practice

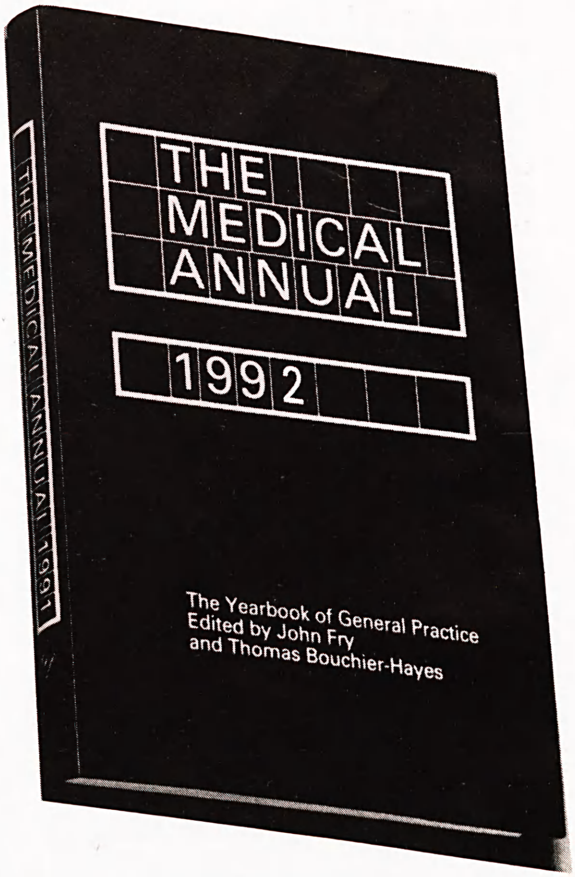

Edited by: Dr. **John Fry**, CBE, MD, FRCS, FRCGP, General Practitioner, President of the General Practice Section of the Royal Society of Medicine, Senior Treasurer General Medical Council, Council Member Royal College of General Practitioners.

Professor **Thomas Bouchier Hayes**, FRCGP, Professor of General Practice, The Royal Army Medical College, Millbank, London.

General Practitioners! Here at your fingertips, in one convenient and easy to use volume, a review of key issues affecting general practice today. Written by leading authorities, each paper provides a comprehensive overview of the latest developments in a particular area whether it be disease, drug therapy or practice management. Just look at the contents to see the varied and interesting coverage of the 1992 issue!

Each issue of the **Medical Annual** reflects current major issues, a subscription will build up into a valuable source of reference for your practice. If you value you time you will value the **Medical Annual.**

READY SOON! ORDER NOW!

**SPECIAL OFFER — SPECIAL OFFER**

To get your collection off to a good start all new subscribers to the **Medical Annual** will receive **FREE** a copy of the 1991 issue while stocks last. So order your subscription NOW!

**Contents:**
Editorial 1991/1992 - GENERAL EVENTS - Medicopolitical Matters - Medicolegal Matters - SIMG: The International Society of General Practice - General Practice in Europe, Australia, New Zealand, Finland and Israel - HEALTH AND DISEASE - Primary Care and Osteoporosis in Women - Progress in the Field of Allergy - Recent Progress and Advances in Haemophilia - Migraine - Recent Progress and Advances in Rheumatism and Arthritis - Sleep Apnoea Syndrome - Travel Medicine - Terminal Care - Paediatric Oncology - Cryosurgery - Post-traumatic Stress Disorder - Epilepsy - Catarrh - Glue Ear; Is Your Grommet Really Necessary? - Cholesterol and Coronary Heart Disease - Stress - THERAPEUTICS - Diabetes Mellitus and its Drug Therapy - Drugs for Rheumatism and Arthritis - Dermatology - Irritable Bowel Syndrome - Helicobacter Pylori - Peptic Ulcer - Oncology - Angiotension-converting Enzyme Inhibitors in Hypertension - PREVENTION AND HEALTH PROMOTION - Care of the Disabled for the Community - Screening: The Benefits? - PRACTICE MANAGEMENT AND ORGANISATION - Medical Audit and Medical Audit Protocols - Teamwork in Practice and the New Contract - The Postgraduate Education Allowance - Organising Meetings - Cost of Medicines and Prescribing - Cost: The Watchword for Prescribing in the 1990's - NEW TECHNOLOGY - New Tools for General Practitioners - MISCELLANY - Facts 1991 - Royal College of General Practitioners - INDEX

*250 pages approx. 30mm x 150mm. Casebound.*
*ISSN: 0076 5899. ISBN: 1 85457 028 5. £25.00*

**International Editorial Board**
Dr. Stephanie A. Amiel
Professor Martin J. Bass (Canada)
Dr. David Brooks
Professor J. O. Drife
Professor Sir Michael Drury, OBE
Professor Harold Ellis, CBE
Dr. Wesley E. Fabb (Australia)
Dr. Lionel Fry
Dr. Eric Gambril
Dr. Martin Godfrey
Miss Pauline Jeffree
Professor Pertti Kekki (Finland)
Dr. Gerald Sandler
Dr. Kenneth Scott
Professor V. Sidel (United States of America)
Dr. Philip H. Sive (Israel)
Professor George Teeling Smith
Professor C. Van Weel (Netherlands)
Professor Kerr L. White
(United States of America)
Dr. Gregory Wilkinson
**With many eminent contributors**

**ORDER FORM**

Please order from your usual bookseller or direct from Clinical Press Ltd., c/o Gazelle Book Services, Falcon House, Queen Square, Lancaster, LA1 1RN, UK

............ Please enter my subscription to the **Medical Annual** starting with the 1992 issue and continuing until cancelled.

Please send me copy(s) of the **Medical Annual 1992** at £25.00. **Prepaid orders or credit-card orders will be supplied post free**

............ I enclose a cheque for .............................. (Payable to Gazelle Book Services)

OR I authorise you to charge my Access/Visa/American Express credit account

Card: .......................................... Number: ........................................ Expiry Date ........................................ Signature: ........................................

CLINICAL PRESS

OR Please invoice me at list price plus £2.50 per copy postage & packing Signature: ........................................

Name: (Please Print) ..........................................................................................

Address: (Please Print) ..........................................................................................

Paul Goddard

### This issue:

In this issue there is a concentration on two themes : Surgery and General Practice. John Farndon provides a fascinating review of advances in surgery as reported in the British Journal of Surgery. This is followed by an article on the ethics of transplantation. Such subjects are important topics for the profession but are too infrequently addressed by the practitioners themselves. I am most pleased, therefore, to publish the viewpoint of Mr. Taylor FRCS, past-president of the British Transplantation Society and head of transplantation at Royal Victoria Infirmary, Newcastle-upon-Tyne. These articles are followed by two articles relating to General Practice and then a mix of abstracts, book reviews, articles, news etc.

We hope that you enjoy this issue. We are intending including a questionnaire in the next issue to obtain YOUR views on what should be in the journal.

P G

### Future issues of WEMJ

The West of England Medical Journal is a peer-review journal cited in Index Medicus. Original scientific articles should be sent to the Editor, WEMJ, Redland Green Farm, Redland, Bristol BS6 7HF.
Publication is dependent on referees opinions, editors' decisions and space in the journal.
Some of the future issues will be themed with particular topics including Chest Medicine, Urology and Acquired Immune Deficiency Syndrome.
Contributions on local medical matters, history of medicine, news, views and obituaries are also sought.

### Also from Clinical Press:

**CLINICAL MRI**

***The practical journal of Magnetic Resonance***

**The second issue is now available!**

This journal will assist newcomers to the technique as well as those who are already experienced. "Clinical MRI" is edited by radiologists who are using MRI clinically and the editors are supported by an international team of radiologists, radiographers and physicists.
Over a three-year period the journal will build into a complete text on MRI covering physics, specifications, installation, finance, sectional anatomy and comprehensive reviews on the use of MRI in all clinical circumstances.

**Subscription rate: only £30 per annum**

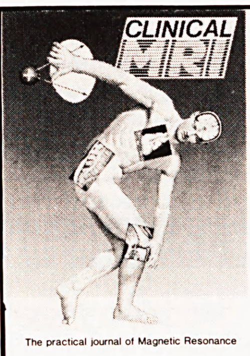

To the Editor

Clinical MRI

Redland Green Farm

Redland

Bristol, BS6 7HF, UK

I wish to subscribe to Clinical MRI at

£30 per annum.

Name ______
Address ______
______
______
Signature ______

**The first title in the CLINICAL EMERGENCY SERIES . . . .**

### ESSENTIAL MANAGEMENT OF OBSTETRIC EMERGENCIES

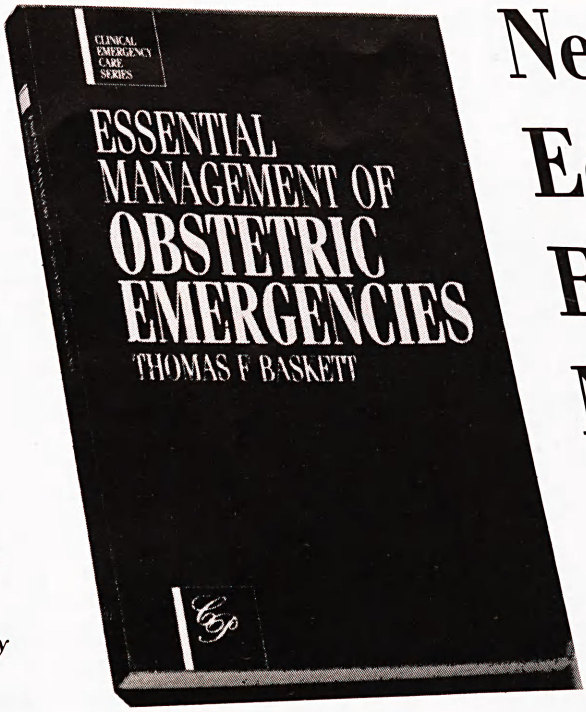

### New Edition Ready Now!

THOMAS F. BASKETT

MB, BCh, BAO (The Queen's University of Belfast)
FRCS(C), FRCS(Ed.), FRCOG, FACOG

*Professor, Department of Obstetrics and Gynaecology*
*Dalhousie University*

*Chief, Department of Gynaecology, Halifax Infirmary*
*Halifax, Nova Scotia, Canada*

**Clear, concise and immensely practical, this up-to-date book is written for those involved in obstetric emergencies at all levels of care — obstetricians, trainees, family practitioners, midwives and nurses. *Essential Management of Obstetric Emergencies* will be especially useful to those who infrequently encounter such problems.**

**The advice, drawn from the author's extensive experience, is authoritative and unequivocal. *Essential Management of Obstetric Emergencies* is divided in twenty-five brief, well-focussed chapters. The book is well illustrated and fully indexed with selected references to further reading.**

**From the Intenational Medical Press**

'It can be recommended for midwives, general practitioners and obstetricians in training who want a clear explanation of present problems and their management.' *Lancet*

'This book is likely to prove popular with obstretricians at all levels but it will be particularly valuable to those in training and they should have ready access to it.' *British Journal of Obstetrics and Gynaecology*

'It belongs on the bookshelf of every library where students work'. *South African Medical Journal*

'The author is to be congratulated on producing a book which lives up to its title by providing clear instructions on how to proceed in any of the common obstetric emergencies.' *Journal of Obstetrics and Gynaecology*

**Contents**

Maternal and Perinatal Mortality - Antenatal Care and Risk Identification - Abortion - Ectopic Pregnancy - Acute Abdominal Pain in Pregnancy - Antepartum Haemorrhage - Sever Pre-eclampsia and Eclampsia - Disseminated Intravascular Coagulation in Pregnancy - Pre-term Labour and Premature Rupture of the Membranes - NON-PROGRESSIVE LABOUR: Dystocia - Shoulder Dystocia - Cord Prolapse - Breech Delivery - Twin Delivery - Fetal Distress in Labour - Uterine Rupture - Complications of the Third Stage of Labour - Post Partum Infection - Post Partum Psychosis - Venous Thromboembolism - Amniotic Fluid Embolism - Haemorrhagic (Hypovolaemic) Shock - OBSTETRIC HAEMORRHAGE: Major Vessel Ligation and Embolization - Analgesia for Emergency Obstetric Procedures - Immediate Care of the Newborn — INDEX

**ORDER YOUR COPY NOW ON THE ORDER FORM PROVIDED**

Paperbound. 225 pages approx.
215mm x 135mm. Fully illustrated.
ISBN: 1 85457 022 6. Only £12.50

**ORDER FORM**

Please order from your usual bookseller or direct from Clinical Press Ltd., c/o Gazelle Book Services, Falcon House, Queen Square, Lancaster, LA1 1RN, UK

Please send me copy(s) of the **Essential Management of Obstetric Emergencies** at £12.50. **Prepaid orders or credit-card orders will be supplied post free**

............ I enclose a cheque for .............................. (Payable to Gazelle Book Services)

OR I authorise you to charge my Access/Visa/American Express credit account

Card: ........................................ Number: ........................................ Expiry Date ........................................ Signature: ........................................

CLINICAL PRESS

OR Please invoice me at list price plus £2.50 per copy postage & packing Signature: ........................................

Name: (Please Print) ........................................

Address: (Please Print) ........................................

**Self assessment radiology**

### Clinical Film Viewing Series

Series Editor: Paul R. Goddard, MD., FRCR

Most postgraduate examinations now include radiographic interpretation. This may be presented as a slide show or as a film-viewing session. Radiographs and other imaging modalities are also used including those of the American Boards and professional societies and universities world wide.

In a typical examination situation, candidates are asked to view a film but are provided with minimal clinical information. They are then asked to give a written or oral report together with a sensible list of possible differential diagnoses.

This series of books on *Clinical Film Viewing* will assist candidates to prepare for this process. Each book examines a particular system or technique and promotes the reader's self-questioning about the film presented. The series provides a unique opportunity for self-assessment and learning in all the modern modalities of diagnostic imaging.

Each book consists of an introduction to the speciality or technique followed by eighty profusely illustrated cases typical of those presented in postgraduate examinations.

---

### THE RESPIRATORY SYSTEM

by PAUL GODDARD, M.D., F.R.C.R.

This book will assist candidates to prepare for the final examination of the F.R.C.R. The book consists of eighty profusely illustrated cases and many lists and flowcharts describing the modern practice of chest radiology. The text is readable and concise.

*This is an invaluable learning book!* ISBN: 1 85457 014 5 **£15.00**

---

### THE CARDIOVASCULAR SYSTEM

by GEORGE HARTNELL, F.R.C.R.

"A well-organized presentation of case material which will help residents in radiology and other specialities to assess their knowledge of radiological diagnosis of cardiovascular disease".

*A. de Roos, M.D., European Journal of Radiology.* ISBN: 1 85457 012 9 **£15.00**

---

**COMING SOON**

### ULTRASOUND by KABALA and SLACK

A comprehensive introduction to ultrasound, illustrated by case presentation.

ISBN: 1 85457 013 7 **£15.00**

---

**ALSO**

### THE RADIOLOGY OF AIDS by A. Banerjee

The protean manifestations of AIDS shown throughout the head and body. Including chest x-rays, barium examinations, CT and MRI. ISBN: 1 85457 025 0.

